# Serum *trans* fatty acids, asymmetric dimethylarginine and risk of acute myocardial infarction and mortality in patients with suspected coronary heart disease: a prospective cohort study

**DOI:** 10.1186/s12944-016-0204-9

**Published:** 2016-02-27

**Authors:** Heidi Borgeraas, Jens Kristoffer Hertel, Reinhard Seifert, Rolf K. Berge, Pavol Bohov, Per Magne Ueland, Ottar Nygård, Jøran Hjelmesæth

**Affiliations:** Morbid Obesity Center, Vestfold Hospital Trust, Tønsberg, Norway; Department of Clinical Science, University of Bergen, Bergen, Norway; Department of Heart Disease, Haukeland University Hospital, Bergen, Norway; Labotatory of Clinical Biochemistry, Haukeland University Hospital, Bergen, Norway; KG Jebsen Center for Diabetes Research, Haukeland University Hospital, Bergen, Norway; Department of Endocrinology, Morbid Obesity and Preventive Medicine, Institute of Clinical Medicine, University of Oslo, Oslo, Norway

**Keywords:** Acute myocardial infarction, Asymmetric dimethylarginine, Cardiovascular disease, Mortality, Trans fatty acid

## Abstract

**Background:**

*Trans* fatty acids (TFAs) have been found to impair flow mediated vasodilation and nitric oxide (NO) production. We sought to examine if serum TFA levels are associated with plasma levels of the NO inhibitor asymmetric dimethylarginine (ADMA) and if possible relationships between serum TFA and cardiovascular morbidity or mortality are mediated or modified by plasma ADMA levels.

**Methods:**

The cohort included patients who underwent coronary angiography for suspected coronary heart disease in 2000–2001. Serum *trans* 16:1n7 and *trans* 18:1 isomers were determined by gas liquid chromatography and the summation of these two TFAs is reported as TFA (percentage by weight (wt%) or concentration). Associations between TFAs and ADMA were estimated by calculating the Spearman’s rank correlation coefficient (ρ), and risk associations with AMI, cardiovascular death and all-cause mortality across quartiles of TFAs (wt% or concentration) were explored by Cox modeling.

**Results:**

A total of 1364 patients (75 % men) with median (25^th^,75^th^ percentile) age 61 (54, 69) years, serum TFA 0.46 (0.36, 0.56) wt% and plasma ADMA 0.59 (0.50, 0.70) μmol/L were studied. Serum TFA levels (ρ = 0.21, *p* < 0.001), *trans* 16:1n7 (ρ = 0.22, *p* < 0.001) and *trans* 18:1 (ρ = 0.20, *p* < 0.001) levels were significantly correlated with plasma ADMA levels. During the median (25^th^,75^th^ percentile) follow-up time of 5.8 (4.5, 6.4) years, 129 (9.5 %) patients experienced an AMI, 124 (9.1 %) died, whereof 66 (53 %) due to cardiovascular causes. After multivariate adjustments no significant associations between serum TFA levels (wt% or concentration) and incident AMI, CV death and all-cause mortality were observed. Similar results were obtained when repeating the analyses with *trans* 16:1n7 and *trans* 18:1 individually. Plasma ADMA levels did not significantly modify the associations between TFA levels and outcomes.

**Conclusions:**

Serum TFA levels were positively correlated with plasma ADMA levels. After multivariate adjustments, TFAs were not associated with incident AMI or mortality, and associations were not influenced by ADMA.

**Trial registration:**

Clinicaltrials.gov Identifier: NCT00354081

**Electronic supplementary material:**

The online version of this article (doi:10.1186/s12944-016-0204-9) contains supplementary material, which is available to authorized users.

## Background

*Trans*fatty acids (TFAs) are naturally present in small quantities in dairy products and meat, but the main dietary source is processed foods containing partially hydrogenated vegetable oils. While no, or even beneficial cardiovascular (CV) effects of TFAs from dairy products and meat have been claimed, a high consumption of industrially produced TFAs have repeatedly been associated with an increased risk of CV disease [1, 2]. The underlying mechanisms of the adverse effects are multifaceted but at present predominantly explained by the influence of TFAs on serum lipid levels [3–5]. Also, TFAs have been found to increase markers of inflammation, promote thrombogenesis [6–8] and impair flow mediated dilatation [9]. More recently, human endothelial cells treated with TFAs were found to have decreased production of the important vasodilator nitric oxide (NO) [10].

Asymmetric dimethylarginine (ADMA) is a potent inhibitor of NO synthesis, and numerous studies have linked increasing levels of ADMA with risk of CV disease [11]. ADMA is metabolized by the enzyme dimethylarginine dimethylamoniohydrolase (DDAH) [12, 13], which is a critical regulator of ADMA levels. DDAH activity is in turn up-regulated by activation of peroxisome proliferator-activated reseptor γ (PPARγ) [14] and inhibition of sterol regulatory element binding protein-1c (SREBP-1c) [15]. TFAs have been found to down-regulate PPARγ mRNA expression [16] and to up-regulate SREBP-1c mRNA expression [17]. Additionally, TFAs are associated with higher levels of inflammatory markers, including TNF-α [18], which in turn may down-regulate DDAH activity [19]; hence TFAs may be a potential inhibitor of ADMA degradation, with a subsequent decrease of NO levels.

As of today, the mechanisms by which TFAs are related to CV disease are not fully understood. Thus, in over 1300 Norwegian patients with suspected coronary heart disease (CHD), we aimed to examine if serum TFA levels (*trans* 16:1n7 and *trans* 18:1 isomers) were associated with plasma ADMA levels, and if potential associations between serum TFA levels and risk of acute myocardial infarction (AMI), CV death and all-cause mortality were mediated or modified by plasma ADMA.

## Methods

### Study population

The Bergen Coronary Angiography Cohort (BECAC) includes 4241 patients undergoing coronary angiography for suspected CHD (stable angina pectoris or acute coronary syndrome (ACS) [20]. The initial 1367 patients recruited to BECAC during 2000–2001 were selected for fatty acid (FA) analyses. Three patients were excluded due to no data on plasma ADMA, leaving 1364 patients eligible for analyses. Of these patients, 707 were also recruited to the Western Norway B-Vitamin Intervention Trial (WENBIT) (ClinicalTrials.gov Identifier: NCT00354081), a RCT investigating the effect of high dose B-vitamin supplementation on risk of CV disease and mortality [21]. The study protocol was approved by the Regional Committee for Medical Research Ethics and the Norwegian Data Inspectorate and met the mandate of the Declaration of Helsinki. All participants signed a consent form.

### Baseline data

As previously reported each patient completed a self-administered questionnaire on medical history, risk factors and medications, and the information was subsequently validated against medical records [20, 21]. Diabetes mellitus included type 1 and 2 as previously diagnosed, independent of serum glucose or HbA1C levels at baseline. Current smoking included self-reported current smoking, those who had quit smoking within the last month and patients with plasma cotinine >85 ng/mL [22]. Fasting referred to abstaining from food at least 6 h prior to blood sample collection. Left ventricular ejection fraction (LVEF) (%) and extent of significant stenosis of the coronary arteries were verified as previously reported [23]. Effective statin dose refers to the ranked value of the expected percent of LDL-cholesterol reduction based on type and dose of specified statin use [24, 25].

### End point and follow up

Clinical endpoints were AMI (fatal and non-fatal), CV death and all-cause mortality. The patients were followed from angiography in 2000 or 2001 until any endpoint occurred, or throughout December 31^th^ 2006.

Collection of clinical events information has previously been described [26]. AMI definition, published in 2000 [27], was used as diagnostic criteria. CV death included death causes coded I00-I99 or R96 (International Statistical Evaluation of Disease, 10^th^ Revision System). Events were classified as fatal if death occurred within 28 days after onset. An endpoints committee adjudicated all events, with no information on baseline characteristics.

### Biochemical analyses

Serum and plasma samples were collected prior to angiography and stored at −80 °C until analysis. ApoA1, ApoB and lipoprotein (a) concentrations were determined using the Hitachi 917 system (Roche Diagnostics, GmbH, Mannheim, Germany). C-reactive protein (CRP) was measured using a latex, high sensitive assay (Behring Diagnostics, Marburg, Germany). Serum was treated with 2 % (v/v) of sulfuric acid in methanol to prepare serum FA methyl esters (FAMEs) [28]. FAMEs were analyzed by gas-liquid chromatography (GC 8000 TOP, Finnigan, USA) on DB1-ms capillary column (J & W Scientific, USA) coupled to a flame-ionization detector [29]. The method is developed for analyses of a general FA profile, and included *trans* 16:1n7 and *trans* 18:1 isomers. Other positional isomers were thus not separated on the column. The two TFAs combined are here referred to as serum TFA (percentage by weight (wt%) or concentration). With-in day coefficient of variation was 2.22 % for TFA (wt%), 3.00 % for *trans* 16:1n7 (wt%) and 2.33 % for the *trans* 18:1 isomers (wt%). Plasma ADMA was measured using high performance liquid chromatography/tandem mass spectrometry (LC-MS/MS) at BEVITAL AS (www.bevital.no), and within-day coefficient of variation was 5–7 %. Cotinine was determined by LC-MS/MS [30]. The Friedewald formula was used to calculate LDL cholesterol, and estimated glomerular filtration rate (eGFR) was calculated using the Chronic Kidney Disease Epidemiology Collaboration [31].

### Statistical methods

Continuous variables are presented as median (25^th^, 75^th^ percentile), unless otherwise stated, and categorical variables as count (percentage). Trends across quartiles of serum TFAs (wt%) and plasma levels of ADMA were calculated using median linear regression for continuous variables and logistic regression for binary variables.

A generalized additive model (GAM) was fitted to assess for possible non-linear relationships between serum TFA (wt%) and plasma ADMA levels. Bivariate correlation analyses between serum TFA, *trans* 16:1n7, *trans* 18:1 (as wt% and concentration) and plasma ADMA levels were calculated as the Spearman’s rank correlation coefficient. Multivariate adjusted correlation analyses between serum TFAs, *trans* 16:1n7, *trans* 18:1 and plasma ADMA levels were calculated as the partial rank correlation coefficient, adjusting for the clinically relevant variables age (years), sex, effective statin dose at baseline (0–6), fasting (yes/no), eGFR (mL/min), ACS diagnosis prior to baseline coronary angiography (yes/no) and serum triglycerides (mmol/L) levels.

Hazard ratios (HR) of AMI, CV death and all-cause mortality according to serum levels of serum TFAs were estimated with Cox proportional hazard models. When evaluating the relationships between quartiles of TFA levels and subsequent risk of AMI or mortality, the first quartile was used as reference. Variables in the multivariate adjusted model were selected based on clinical relevance and included age (years), sex, diabetes mellitus (yes/no), current smoking (yes/no), effective statin dose at discharge (0–6), extent of significant stenosis of the coronary arteries (0–3) and eGFR (mL/min)). WENBIT intervention status (vitamin B6 (yes/no) or folate/B12 (yes/no)) had no effect on any estimates (data not shown), and was not included in the model.

Additionally, the association between ADMA and risk of AMI, CV death and all-cause mortality was examined in a Cox model (adjusting for age and sex and also TFA (wt%)) to confirm a relationship between the possible mediator and outcome independent of the exposure (TFA). An additional GAM model was fitted to assess for non-linear relationships.

Potential mediation by ADMA was examined by adding ADMA (μmol/L) to the univariate, age and sex and multivariate adjusted Cox models examining the effect of TFA on outcomes. Possible effect modification was explored by including an interaction product term of TFA (quartiles) and ADMA (above and below median) in the same Cox models.

All probability values are 2-tailed, and were considered significant when <0.05. Statistical analyses were performed with SPSS 21 (SPSS Inc, Chicago, IL) and R 2.14.2 (The R-Foundation for Statistical Computing, Vienna, Austria).

## Results

### Demographic and clinical characteristics

Baseline clinical characteristics of the 1364 participants, according to quartiles of serum TFA levels (wt%), are presented in (Table 1). In the total population, wt% and concentration of serum TFA levels ranged between 0.10–1.61 % and 2,69–255 mg/L, respectively.

**Table 1:** Baseline characteristics of participants by quartiles of serum *trans* fatty acids

		Quartiles of serum *trans* fatty acids (wt%)				
	Total	Q1	Q2	Q3	Q4	
		0.30 (0.10, 0.36)^a^	0.41 (0.37, 0.45)	0.51 (0.46, 0.55)	0.64 (0.56, 1.61)	
	*n* = 1364	*n* = 355	*n* = 325	*n* = 331	*n* = 353	p-trend^b^
Demographic and clinical characteristics						
Male sex^c^	1019 (74.7)	296 (83.4)	240 (73.8)	239 (72.2)	244 (69.1)	<0.001
Age (years)^d^	61 (54, 69)	57 (51, 63)	61 (54, 68)	64 (56, 71)	65 (55, 72)	<0.001
BMI (kg/m^2^)	26.2 (24.1, 28.7)	26.7 (24.4, 29.0)	26.0 (24.3, 28.7)	26.1 (24.0, 28.7)	26.1 (23.8, 28.7)	0.11
Fasting	187 (14.5)	69 (20.7)	46 (15.0)	34 (10.7)	38 (11.4)	<0.001
Diastolic blood pressure (mmHg)	80 (75, 88)	81 (75, 90)	80 (75, 87)	80 (74, 87)	80 (74, 89)	1.00
Systolic blood pressure (mmHg)	138 (125, 152)	139 (126, 154)	139 (125, 150)	136 (124, 150)	137 (122, 154)	0.08
Cardiovascular history						
Previous AMI	553 (40.5)	140 (39.4)	126 (38.8)	137 (41.4)	150 (42.5)	0.84
Previous PCI	239 (17.5)	83 (23.4)	61 (18.8)	45 (13.6)	50 (14.2)	<0.01
Previous CABG	134 (9.8)	32 (9.0)	38 (11.7)	33 (10.0)	31 (8.8)	0.70
Risk factors						
Impaired ejection fraction	151 (11.1)	42 (11.8)	30 (9.2)	38 (11.5)	41 (11.6)	0.98
Diabetes	140 (10.3)	38 (10.7)	26 (8.0)	33 (10.0)	43 (12.2)	0.71
Current smoker	457 (33.5)	158 (44.5)	101 (31.1)	92 (27.8)	106 (30.0)	0.05
Clinical diagnosis before baseline coronary angiography						
Stable angina	1273 (93.3)	318 (89.6)	296 (91.1)	315 (95.2)	344 (97.5)	<0.001
Acute coronary syndrome	91 (6.7)	37 (10.4)	29 (8.9)	16 (4.8)	9 (2.5)	<0.001
Extent of coronary artery stenosis at baseline coronary angiography						
No significant stenosis	234 (17.2)	44 (12.4)	67 (20.6)	58 (17.5)	65 (18.4)	0.09
1-vessel disease	368 (27.0)	134 (37.7)	74 (22.8)	85 (25.7)	75 (21.2)	<0.001
2-vessel disease	329 (26.3)	91 (25.6)	82 (25.2)	94 (28.4)	92 (26.1)	0.35
3-vesseldisease	403 (29.5)	86 (24.2)	102 (31.4)	94 (28.4)	121 (34.3)	0.04
Medication following baseline coronary angiography						
Acetylsalisylic acid	1175 (86.1)	316 (89.0)	276 (84.9)	284 (85.8)	299 (84.7)	0.14
ADP receptor blocker	322 (23.6)	118 (33.2)	75 (23.1)	60 (18.1)	69 (19.5)	<0.01
Statins	1107 (81.2)	309 (87.0)	263 (80.9)	255 (77.0)	280 (79.3)	0.01
β-blockers	1022 (74.9)	268 (75.5)	235 (72.5)	248 (74.9)	271 (77.0)	0.43
ACE inhibitors	272 (19.9)	75 (21.1)	64 (19.7)	60 (18.1)	73 (20.7)	0.36
Loop diuretika	145 (10.6)	26 (7.3)	32 (9.8)	42 (12.7)	45 (12.7)	0.11
Fatty acids						
Total fatty acids (mg/L)	3932 (3320, 4673)	3913 (3225, 4646)	3881 (3345, 4490)	3883 (3293, 4570)	4114 (3407, 4835)	0.02
*Trans* fatty acid (mg/L)	17.8 (13.3, 23.8)	11.5 (8.97, 14.0)	15.8 (13.3, 18.7)	19.6 (16.5, 23.1)	26.9 (22.3, 33.8)	<0.001
*Trans* 16:1n7 (mg/L)	2.64 (1.91, 3.68)	1.75 (1.36, 2.24)	2.46 (1.89, 3.09)	2.95 (2.40, 3.77)	3.81 (2.93, 4.94)	<0.001
*Trans* 18:1 (mg/L)	15.0 (11.2, 20.2)	9.66 (7.57, 11.8)	13.3 (11.2, 15.6)	16.4 (14.0, 19.7)	22.9 (18.8, 29.1)	<0.001
Lipid related parameters						
ApoA1 (g/L)	1.29 (1.14, 1.46)	1.31 (1.17, 1.49)	1.31 (1.14, 1.48)	1.28 (1.13, 1.43)	1.26 (1.12, 1.45)	<0.01
ApoB (g/L)	0.91 (0.76, 1.07)	0.89 (0.76, 1.06)	0.91 (0.76, 1.07)	0.91 (0.74, 1.08)	0.92 (0.78, 1.07)	0.16
Cholesterol (mmol/L)	5.00 (4.30, 5.80)	5.00 (4.30, 5.70)	5.10 (4.40, 5.95)	5.00 (4.30, 5.90)	5.10 (4.30, 5.80)	0.33
HDL Cholesterol (mmol/L)	1.20 (1.00, 1.43)	1.20 (1.00, 1.40)	1.20 (1.00, 1.50)	1.20 (1.00, 1.40)	1.17 (0.94, 1.40)	1.00
Triglycerides (mmol/L)	1.52 (1.13, 2.15)	1.44 (1.08, 2.18)	1.48 (1.13, 1.92)	1.49 (1.08, 2.02)	1.67 (1.20, 2.40)	0.01
Lp(a) (mmol/L)	0.21 (0.10, 0.54)	0.25 (0.11, 0.59)	0.22 (0.10, 0.54)	0.19 (0.09, 0.48)	0.22 (0.10, 0.54)	0.11
Other parameters						
Glucose (mmol/L)	5.70 (5.10, 6.70)	5.80 (5.10, 6.80)	5.60 (5.10, 6.50)	5.60 (5.20, 6.50)	5.70 (5.00, 6.80)	1.00
HbA1c (mmol/L)	5.84 (5.17, 6.54)	5.77 (5.19, 6.38)	5.77 (5.12, 6.44)	5.95 (5.22, 6.65)	5.87 (5.09, 6.73)	0.11
Arginine (μmol/L)	71.5 (50.9, 90.5)	77.8 (56.0, 96.5)	70.4 (51.2, 87.7)	65.3 (50.2, 88.2)	71.6 (49.1, 91.1)	<0.01
ADMA (μmol/L)	0.59 (00.50, 0.70)	0.54 (0.48, 0.65)	0.57 (0.49, 0.68)	0.61 (0.52, 0.74)	0.61 (0.52, 0.74)	<0.001
eGFR (mL/min)	91.0 (78.0, 99.0)	96.0 (85.0, 103)	90.0 (79.0, 99.0)	89.0 (77.0, 98.0)	87.0 (70.5, 97.5)	<0.001
CRP (mg/L)	1.92 (0.95, 3.98)	2.18 (1.00, 4.28)	1.82 (0.89, 3.91)	1.82 (0.97, 3.66)	1.97 (1.00, 3.77)	0.40

*Abbreviations*: *ApoA-1* apolipoprotein A-1, *BCA* baseline coronary angiography, *BMI* body mass index, *CRP* C-reactive protein, *GFR* glomerular filtration rate, *HbA1c* hemoglobin A1c, *HDL* high density lipoprotein, *LDL* low density lipoprotein, *Lp(a)* lipoprotein(a), *PCI* percutanous coronary intervention, *wt%* percentage by weight

^a^Median (range)

^b^Median linear regression for continuous and logistic regression for categorical variables

^c^Count (percentage) for all such values

^d^Median (25^th^, 75^th^ percentile) for all such values

Patients with higher serum TFA levels were characterized by older age, a relatively higher proportion of females and non-fasting patients. They had less often undergone PCI and were less often diagnosed with ACS prior to baseline coronary angiography and 1-vessel disease following baseline coronary angiography. Also, they were less frequently discharged with statins and ADP-receptor blockers. However, they were more often diagnosed with 3-vessel disease following baseline coronary angiography.

Patients with higher serum TFA levels had higher plasma ADMA levels, serum triglyceride and total FA levels and lower eGFR and serum ApoA-I levels.

### Correlations between TFAs and ADMA

Bivariate and multivariate correlation analyses revealed highly significant (*p* < 0.001) and positive relationships between serum levels of TFAs and of the individual TFAs (both as wt% and concentration) and plasma ADMA levels (ρ = 0.12–0.22) (Table 2), and the GAM plot revealed no non-linear associations (data not shown). The estimates were not significantly influenced by additional adjustments for previous PCI, current smoking, extent of significant stenosis of the coronary arteries or plasma arginine.

**Table 2:** Correlations^a^ between *trans* fatty acids and ADMA

	Bivariate	Multivariate^b^	Multivariate^c^
Percentage by weight (wt%)			
*Trans* fatty acid	0.21	0.16	0.14
*Trans* 16:1n7	0.22	0.16	0.12
*Trans* 18:1	0.20	0.15	0.14
Concentration (mg/L)			
*Trans* fatty acid	0.18	0.15	0.20
*Trans* 16:1n7	0.21	0.17	0.18
*Trans* 18:1	0.17	0.15	0.20

^a^ Spearman’s ranked (partial) correlation coefficient (ρ)

^b^ Adjusted for age (years) and sex

^c^ Adjusted for age (years), sex, effective statin dose at baseline (0–6), fasting (yes/no), eGFR (mL/min), ACS (yes/no), triglycerides (mmol/L)

All correlation are significant at the 0.001 level (2-tailed)

The correlation between serum total FA levels and plasma ADMA levels (adjusted for TFA concentration, age and sex) was inverse (ρ = −0.06, *p* = 0.018).

### Serum TFA levels and risk of AMI, CV death and all-cause mortality

During the median (25^th^, 75^th^ percentile) follow-up period 5.8 (4.5, 6.4) years), 129 (9.5 %) patients had an incident AMI, of which 44 were fatal (34 %). A total of 124 (9.1 %) patients died, whereof 66 (53 %) deaths were due to CV causes.

In univariate analyses, there were significant trends of increased risk of CV death and all-cause mortality across quartiles of TFA (Table 3). However, after multivariate adjustments no significant associations between TFA (wt% or concentration) and endpoints were observed. Similar results were obtained when repeating the analyses with *trans* 16:1n7 (Additional file 1: Table S1) and *trans* 18:1 (Additional file 2: Table S2) (wt% and concentration) individually. Further adjustments for BMI, blood pressure, fasting, impaired LVEF, previous PCI, ACS diagnosis prior to baseline coronary angiography, use of ADP receptor blocker, use of loop diuretics or concentration of any lipid related parameter did not significantly influence the results. Additionally, GAM plots revealed no non-linear associations (data not shown).

**Table 3:** *Trans* fatty acid quartiles and risk of acute myocardial infarction, cardiovascular death and all-cause mortality

			Percentage by weight (wt%)						Concentration (mg/L)			
Model	AMI		CV death		All-cause mortality		AMI		CV death		All-cause mortality	
	HR	95 % CI	HR	95 % CI	HR	95 % CI	HR	95 % CI	HR	95 % CI	HR	95 % CI
Univariate												
Q2	1.13	0.66, 1.93	1.69	0.74, 3.87	1.29	0.71, 2.36	1.10	0.66, 1.83	1.05	0.51, 2.15	0.96	0.55, 1.69
Q3	1.49	0.90, 2.45	1.92	0.86, 4.27	1.73	0.99, 3.04	1.05	0.63, 1.75	0.74	0.34, 1.60	0.95	0.54, 1.65
Q4	1.36	0.82, 2.25	2.39	1.11, 5.14	2.24	1.32, 3.82	1.25	0.77, 2.05	1.53	0.79, 2.96	1.84	1.13, 3.00
*P-trend*	0.15		0.03		0.03		0.42		0.28		0.01	
*P-interaction* ^*a*^	0.52		0.74		0.85		0.68		0.38		0.59	
Age and sex adjusted												
Q2	1.07	0.63, 1.84	1.36	0.59, 3.13	1.09	0.60, 2.01	1.11	0.67, 1.85	1.00	0.48, 2.04	0.94	0.54, 1.66
Q3	1.31	0.89, 2.18	1.23	0.54, 2.77	1.21	0.69, 2.15	1.02	0.61, 1.70	0.63	0.29, 1.38	0.85	0.49, 1.49
Q4	1.19	0.71, 2.00	1.44	0.65, 3.18	1.51	0.87, 2.62	1.25	0.76, 2.05	1.36	0.69, 2.65	1.73	1.06, 2.85
*P-trend*	0.41		0.45		0.10		0.46		0.49		0.02	
*P-interaction* ^*a*^	0.68		0.48		0.74		0.64		0.29		0.51	
Multivariate adjusted^b^												
Q2	1.10	0.64, 1.90	1.43	0.62, 3.31	1.18	0.64, 2.17	1.08	0.65, 1.80	1.00	0.48, 2.07	0.97	0.55, 1.71
Q3	1.43	0.86, 2.39	1.36	0.60, 3.09	1.36	0.76, 2.41	1.02	0.61, 1.72	0.62	0.28, 1.37	0.89	0.50, 1.56
Q4	1.14	0.67, 1.92	1.39	0.62, 3.11	1.53	0.87, 2.67	1.11	0.67, 1.84	1.11	0.56, 2.21	1.54	0.93, 2.55
*P-trend*	0.49		0.54		0.11		0.74		0.92		0.08	
*P-interaction* ^*a*^	0.64		0.72		0.80		0.77		0.41		0.74	

The risk associations between TFA, *trans* 16:1n7, *trans* 18:1 and endpoints did not significantly differ between patients who were discharged with statins and those who were not (data not shown).

### Plasma ADMA levels and risk of AMI, CV death and all-cause mortality

Plasma ADMA levels (μmol/L) were positively associated with risk of AMI (HR (95 % CI) (3.44 (1.24, 9.51) *p*-value = 0.017), CV death (7.17 (2.13, 24.2) *p*-value = 0.001) and all-cause mortality (8.04 (3.35, 19.3) *p*-value <0.001) (wt%) (adjusting for age and sex). The associations were only slightly attenuated after an additional adjustment for TFA (wt%): 3.26 (1.16, 9.17) *p*-value = 0.025 for AMI, 7.09 (2.09, 24.0) *p*-value = 0.002 for CV death and 7.45 (3.09, 18.0) *p*-value <0.001 for all-cause mortality. Additionally, GAM plots revealed a linear and positive association between plasma ADMA levels and endpoints (data not shown).

### Mediating and modifying effect of plasma ADMA

Including plasma ADMA (μmol/L) in the univariate and age and sex adjusted Cox models slightly attenuated the effect of TFA on endpoints, but plasma ADMA did not significantly influence the multivariate adjusted estimates (Additional file 3: Table S3). Moreover, the effect of TFA, as either wt% or concentration, on risk of AMI, CV death and all-cause mortality was not modified by ADMA levels (above or below median) (All p for interaction >0.29) (Table 3).

## Discussion

In this prospective cohort study of 1364 Norwegian patients with suspected CHD, there was a positive and significant association between serum TFA levels and plasma ADMA levels. Serum levels of TFAs were associated with increased risk of CV death and all-cause mortality, but not AMI. The associations were, however, attenuated and no longer significant in multivariate models. Plasma ADMA levels were positively related to incident AMI, CV and all-cause mortality, independent of TFA levels, but did not significantly influence the associations between TFA levels and outcomes.

### TFAs and ADMA

As recently reviewed [1], there are numerous studies relating TFAs to adverse effects on CV health, and several possible mechanisms may explain this association. TFAs have been found to unfavorably alter the lipid profile by increasing LDL cholesterol, Lp(a) and triglyceride levels and decreasing HDL cholesterol levels [3–5]. In the current study, we did not observe an association between TFA levels and HDL cholesterol, LDL cholesterol or Lp(a) levels, but in accordance with previous studies [3], patients with higher serum TFA levels had higher circulating levels of triglycerides. Statins alter the lipid profile and were used by the majority of patients in this study. Although adjusted for in all multivariate analyses, use of statins may possibly serve as an explanation as to why we did not observe any significant associations between serum TFA and cholesterol levels. In addition to the modifying effect of TFAs on the lipid profile, dietary intake of TFAs has been associated with an increase in markers of endothelial dysfunction such as CRP, interleukin-6, soluble tumor necrosis factor receptor 2, E-selectin and soluble cell adhesion molecules [7]. We did not, however, observe an association between serum TFA levels and CRP, which may be due to the CRP lowering effect of statins [32]. A previous crossover study among healthy men and women found consumption of TFAs, relative to consumption of saturated FAs, to be associated with reduced flow-mediated vasodilatation (FMD) [9]. Moreover, a recent study found human endothelial cells treated with TFAs to produce less NO [10]. To the best of our knowledge, this is the first study to examine the relationship between serum levels of TFA and the NO inhibitor ADMA. The association was not attenuated by adjustments for other groups of FAs (cis-monounsaturated FAs, omega-3 FAs, omega-6 FAs or saturated FAs) (data not shown). Additionally, the correlation between serum total FA levels and plasma ADMA were inverse and weak, suggesting that the correlation between serum TFAs and plasma ADMA was not caused by high serum FA levels per se. Previously, a positive association between ADMA levels and LDL cholesterol has been observed [19]. However, the reported association between ADMA and TFA was not mediated or confounded by LDL cholesterol, as inclusion of LDL cholesterol in the correlation analyses did not change the results.

### TFAs and incident AMI and mortality

Despite the observed relationships between serum levels of TFAs and markers of endothelial dysfunction, we did not observe any significant associations between TFA and incident AMI or mortality in multivariate adjusted analyses. In this study, two different TFAs were measured in serum; *trans* 16:1n7 and *trans* 18:1. *Trans* 16:1n7 (trans palmitoleic acid) is a natural TFA, but the trans 18:1 pool was most likely a mixture of industrially produced and natural TFAs as the different positional isomers were not separated. Dietary intake of industrially produced TFAs has repeatedly been linked with increased risk of CV disease, whereas there is no clear consensus regarding intake of ruminant TFAs and risk of CV disease [1, 2]. However, in the present study, individually, neither *trans* 16:1n7 nor *trans* 18:1, were associated with incident AMI or mortality, and the associations did not differ between patients with low (below median) or high (above median) plasma ADMA levels. Further, the lack of association between TFA and endpoints even at high or low ADMA levels implies that the excess risk associated with elevated ADMA was explained by other determinants of ADMA levels.

### Strengths and limitations

This study was based on a large, well characterized population and had a prospective design. The relationships between serum TFA levels, plasma ADMA levels and risk of AMI, CV death and all-cause mortality were calculated with TFAs as both wt% and concentration. The wt% of single FAs, or groups of FAs, is influenced by the changes in other FAs, whereas the individual concentrations of FAs are independent of each other. Reporting on both strengthens the validity of our results. Serum levels of TFA reflect dietary intake as these FAs are not synthesized endogenously. However, TFA serum concentration is largely influenced by the FA composition of the latest meals ingested, and tissue concentrations are thought to better reflect the long term dietary FA intake. Thus by only including serum levels in the present study, the risk-associations may have been underestimated. Other limitations include the single baseline measurement of TFAs and biomarkers, which may underestimate associations through regression dilution bias [33]. By the same token, the method by which TFAs were analyzed was not ideal, as there may have been a slight risk of omega-3 FAs co-chromatographing with TFAs. Including omega-3 FAs in the Cox regression analyses did not, however, alter any results (data not shown). Finally, given a low event rate, the statistical power was low.

## Conclusion

In the current study of Norwegian men and women undergoing coronary angiography for suspected CHD there was a positive correlation between serum TFA levels and plasma ADMA levels. We did observe significant positive associations between serum TFA levels and CV death and all-cause mortality in simple models; however after adjustment for possible confounders, no significant associations were detected. Plasma ADMA levels had no significant influence on the observed associations between TFA and outcomes. Further studies are needed to explore the possible underlying mechanisms and consequences of the observed association between serum TFA and plasma ADMA levels.

## Additional files

Additional file 1: Table S1. Quartiles of *trans* 16:1n7 and risk of acute myocardial infarction, cardiovascular death and all-cause mortality. (DOCX 20 kb) Additional file 2: Table S2. Quartiles of *trans* 18:1 and risk of acute myocardial infarction, cardiovascular death and all-cause mortality. (DOCX 20 kb) Additional file 3: Table S3. Quartiles of *trans* fatty acids and risk of acute myocardial infarction, cardiovascular death and all-cause mortality adjusted for plasma ADMA levels. (DOCX 20 kb)
