# Supplementary material for: Serum trans fatty acids, asymmetric dimethylarginine and risk of acute myocardial infarction and mortality in patients with suspected coronary heart disease: a prospective cohort study
Source: Lipids Health Dis. 2016 Feb 27;15:38. doi: 10.1186/s12944-016-0204-9 (PMC4769542; doi:10.1186/s12944-016-0204-9)
Supplement: Additional file 1: Table S1. — Quartiles of trans 16:1n7 and risk of acute myocardial infarction, cardiovascular death and all-cause mortality. (DOCX 20 kb) [file 12944_2016_204_MOESM1_ESM.docx]

| **Additional table 1: Quartiles of *trans* 16:1n7 and risk of acute myocardial infarction, cardiovascular death and all-cause mortality** | | | | | | | | | | | | | | | | | | | | | | | | | |
| --- | --- | --- | --- | --- | --- | --- | --- | --- | --- | --- | --- | --- | --- | --- | --- | --- | --- | --- | --- | --- | --- | --- | --- | --- | --- |
|  | |  | | | | | | | | | | |  | |  | | | | | | | | | | |
|  | | **Percentage by weight (wt%)** | | | | | | | | | | |  | | **Concentration (mg/L)** | | | | | | | | | | |
| **Model** | | **AMI** |  | | **CV death** | | | | | **All-cause mortality** | | |  | | **AMI** | | | | **CV death** | | | | **All-cause mortality** | | |
|  | | HR | 95% CI | | HR | | 95% CI | | | HR | 95% CI | |  | | HR | 95% CI | | | HR | | 95% CI | | HR | | 95% CI |
| **Univariate** | |  |  | |  | |  | | |  |  | |  | |  |  | | |  | |  | |  | |  |
| Q2 | | 1.31 | 0.78, 2.20 | | 1.17 | | 0.50, 2.74 | | | 0.99 | 0.51, 1.19 | |  | | 1.01 | 0.60, 1.70 | | | 1.01 | | 0.45, 2.29 | | 0.93 | | 0.51, 1.70 |
| Q3 | | 1.00 | 0.61, 1.64 | | 1.44 | | 0.70, 2.96 | | | 1.57 | 0.93, 2.66 | |  | | 1.42 | 0.87, 2.31 | | | 1.82 | | 0.88, 3.76 | | 1.75 | | 1.03, 2.97 |
| Q4 | | 1.19 | 0.76, 1.88 | | 1.85 | | 0.95, 3.59 | | | 1.97 | 1.21, 3.20 | |  | | 1.22 | 0.73, 2.02 | | | 1.82 | | 0.88, 3.78 | | 1.93 | | 1.14, 3.26 |
| *P-trend* |  | 0.64 | |  | | 0.056 | |  | <0.01 | |  | | | 0.25 | | |  | 0.04 | |  | | <0.01 | |  |  |
| **Age and sex adjusted** | | |  | |  | |  | | |  | |  |  | |  |  | | |  | |  | |  | |  |
| Q2 | | 1.19 | 0.71, 2.00 | | 0.85 | | 0.36, 2.00 | | | 0.77 | | 0.40, 1.49 |  | | 0.97 | 0.57, 1.64 | | | 0.84 | | 0.37, 1.91 | | 0.81 | | 0.44, 1.49 |
| Q3 | | 0.87 | 0.53, 1.45 | | 0.90 | | 0.43, 1.90 | | | 1.10 | | 0.64, 1.89 |  | | 1.27 | 0.77, 2.09 | | | 1.24 | | 0.59, 2.59 | | 1.30 | | 0.76, 2.23 |
| Q4 | | 0.94 | 0.58, 1.52 | | 0.93 | | 0.46, 1.86 | | | 1.13 | | 0.68, 1.88 |  | | 1.09 | 0.65, 1.83 | | | 1.16 | | 0.55, 2.47 | | 1.37 | | 0.80, 2.35 |
| *P-trend* | | 0.60 |  | | 0.91 | |  | | | 0.41 | |  |  | | 0.52 |  | | | 0.46 | |  | | 0.08 | |  |
| **Multivariate adjusted^a^** | | |  | |  | |  | | |  | |  |  | |  |  | | |  | |  | |  | |  |
| Q2 | | 1.26 | 0.75, 2.12 | | 0.88 | | 0.37, 2.10 | | | 0.78 | | 0.40, 1.53 |  | | 0.97 | 0.57, 1.65 | | | 0.85 | | 0.37, 1.96 | | 0.85 | | 0.46, 1.57 |
| Q3 | | 0.82 | 0.50, 1.37 | | 0.87 | | 0.41, 1.84 | | | 1.07 | | 0.62, 1.83 |  | | 1.30 | 0.79, 2.15 | | | 1.30 | | 0.62, 2.74 | | 1.40 | | 0.81, 2.40 |
| Q4 | | 0.93 | 0.57, 1.51 | | 0.93 | | 0.46, 1.89 | | | 1.15 | | 0.69, 1.94 |  | | 1.03 | 0.61, 1.75 | | | 1.09 | | 0.50, 2.34 | | 1.35 | | 0.78, 2.33 |
| *P-trend* | | 0.49 |  | | 0.89 | |  | | | 0.38 | |  |  | | 0.66 |  | | | 0.59 | |  | | 0.11 | |  |
| Abbreviations: AMI, acute myocardial infarction; CI, confidence interval; CV, cardiovascular; HR, hazard ratio; Q, quartile; wt%, percentage by weight | | | | | | | | | | | | | | | | | | | | | | |  | |  |
| a Adjusted for age (years), sex, current smoking (yes/no), diabetes mellitus (yes/no), effective statin dose at discharge (0-6), extent of significant coronary artery stenosis (0-3) and eGFR (mL/min) | | | | | | | | | | | | | | | | | | | | | | | | | |
