# Supplementary material for: Serum trans fatty acids, asymmetric dimethylarginine and risk of acute myocardial infarction and mortality in patients with suspected coronary heart disease: a prospective cohort study
Source: Lipids Health Dis. 2016 Feb 27;15:38. doi: 10.1186/s12944-016-0204-9 (PMC4769542; doi:10.1186/s12944-016-0204-9)
Supplement: Additional file 2: Table S2. — Quartiles of trans 18:1 and risk of acute myocardial infarction, cardiovascular death and all-cause mortality. (DOCX 20 kb) [file 12944_2016_204_MOESM2_ESM.docx]

| **Additional table 2: Quartiles of *trans* 18:1 and risk of acute myocardial infarction, cardiovascular death and all-cause mortality** | | | | | | | | | | | | | |  |
| --- | --- | --- | --- | --- | --- | --- | --- | --- | --- | --- | --- | --- | --- | --- |
|  |  | | | | | |  |  | | | | | | |
|  | **Percentage by weight (wt%)** | | | | | |  | **Concentration (mg/L)** | | | | | | |
| **Model** | **AMI** | | **CV death** | | **All-cause mortality** | |  | **AMI** | | **CV death** | | **All-cause mortality** | | |
|  | HR | 95% CI | HR | 95% CI | HR | 95% CI |  | HR | 95% CI | HR | 95% CI | HR | 95% CI | |
| **Univariate** |  |  |  |  |  |  |  |  |  |  |  |  |  | |
| Q2 | 1.01 | 0.60, 1.70 | 1.01 | 0.45, 2.89 | 0.93 | 0.51, 1.70 |  | 1.23 | 0.74, 2.03 | 1.30 | 0.63, 2.64 | 1.06 | 0.61, 1.86 | |
| Q3 | 1.42 | 0.87, 2.31 | 1.82 | 0.88, 3.76 | 1.75 | 1.03, 2.97 |  | 1.11 | 0.67, 1.86 | 0.96 | 0.45, 2.04 | 1.13 | 0.65, 1.95 | |
| Q4 | 1.22 | 0.73, 2.02 | 1.82 | 0.88, 3.78 | 1.93 | 1.14, 3.26 |  | 1.28 | 0.78, 2.10 | 1.47 | 0.74, 2.94 | 1.84 | 1.11, 3.03 | |
| *P-trend* | 0.25 |  | 0.04 |  | <0.01 |  |  | 0.44 |  | 0.42 |  | 0.01 |  | |
| **Age and sex adjusted** | |  |  |  |  |  |  |  |  |  |  |  |  | |
| Q2 | 0.97 | 0.57, 1.64 | 0.84 | 0.37, 1.91 | 0.81 | 0.44, 1.49 |  | 1.22 | 0.74, 2.03 | 1.19 | 0.58, 2.43 | 1.01 | 0.58, 1.77 | |
| Q3 | 1.27 | 0.77, 2.09 | 1.24 | 0.59, 2.59 | 1.30 | 0.76, 2.23 |  | 1.09 | 0.65, 1.83 | 0.84 | 0.39, 1.80 | 1.04 | 0.60, 1.81 | |
| Q4 | 1.09 | 0.65, 1.83 | 1.16 | 0.55, 2.47 | 1.37 | 0.80, 2.35 |  | 1.26 | 0.77, 2.09 | 1.29 | 0.64, 2.60 | 1.71 | 1.03, 2.83 | |
| *P-trend* | 0.52 |  | 0.45 |  | 0.08 |  |  | 0.47 |  | 0.66 |  | 0.03 |  | |
| **Multivariate adjusted^a^** | |  |  |  |  |  |  |  |  |  |  |  |  | |
| Q2 | 0.97 | 0.57, 1.65 | 0.85 | 0.37, 1.96 | 0.85 | 0.46, 1.57 |  | 1.23 | 0.74, 2.05 | 1.29 | 0.62, 2.65 | 1.09 | 0.62, 1.93 | |
| Q3 | 1.30 | 0.79, 2.15 | 1.30 | 0.62, 2.74 | 1.40 | 0.81, 2.40 |  | 1.10 | 0.65, 1.85 | 0.84 | 0.39, 1.83 | 1.10 | 0.63, 1.92 | |
| Q4 | 1.03 | 0.61, 1.75 | 1.09 | 0.62, 2.74 | 1.35 | 0.78, 2.33 |  | 1.17 | 0.70, 1.94 | 1.11 | 0.54, 2.27 | 1.58 | 0.94, 2.64 | |
| *P-trend* | 0.65 |  | 0.59 |  | 0.11 |  |  | 0.69 |  | 0.94 |  | 0.08 |  | |
| Abbreviations: AMI, acute myocardial infarction; CI, confidence interval; CV, cardiovascular; HR, hazard ratio; Q, quartile; wt%, percentage by weight | | | | | | | | | | | |  |  | |
| a Adjusted for age (years), sex, current smoking (yes/no), diabetes mellitus (yes/no), effective statin dose at discharge (0-6), extent of significant coronary artery stenosis (0-3) and eGFR (mL/min) | | | | | | | | | | | | | | |
