# Supplementary material for: Serum trans fatty acids, asymmetric dimethylarginine and risk of acute myocardial infarction and mortality in patients with suspected coronary heart disease: a prospective cohort study
Source: Lipids Health Dis. 2016 Feb 27;15:38. doi: 10.1186/s12944-016-0204-9 (PMC4769542; doi:10.1186/s12944-016-0204-9)
Supplement: Additional file 3: Table S3. — Quartiles of trans fatty acids and risk of acute myocardial infarction, cardiovascular death and all-cause mortality adjusted for plasma ADMA levels. (DOCX 20 kb) [file 12944_2016_204_MOESM3_ESM.docx]

| **Additional table 3: Quartiles of *trans* fatty acids and risk of acute myocardial infarction, cardiovascular death and all-cause mortality adjusted for plasma ADMA levels** | | | | | | | | | | | | | | | | |
| --- | --- | --- | --- | --- | --- | --- | --- | --- | --- | --- | --- | --- | --- | --- | --- | --- |
|  |  |  |  | |  | |  |  |  |  |  |  |  |  | |  |
|  | **Percentage by weight (wt%)** | | | | | | | |  | **Concentration (mg/L)** | | | | | | |
|  | **AMI** | | **CV death** | | | | **All-cause mortality** | |  | **AMI** | | **CV death** | | **All-cause mortality** | | |
|  | HR | 95% CI | HR | | 95% CI | | HR | 95% CI |  | HR | 95% CI | HR | 95% CI | HR | | 95% CI |
| **Model 1^a^** |  |  |  | |  | |  |  |  |  |  |  |  |  | |  |
| Q2 | 1.10 | 0.65, 1.88 | 1.59 | | 0.69, 3.63 | | 1.21 | 0.66, 2.22 |  | 1.05 | 0.63, 1.74 | 0.94 | 0.46, 1.94 | 0.87 | | 0.50, 1.52 |
| Q3 | 1.36 | 0.82, 2.26 | 1.60 | | 0.72, 3.58 | | 1.46 | 0.83, 2.56 |  | 0.96 | 0.58, 1.61 | 0.59 | 0.27, 1.29 | 0.76 | | 0.43, 1.34 |
| Q4 | 1.24 | 0.75, 2.07 | 1.96 | | 0.90, 4.26 | | 1.85 | 1.08, 3.17 |  | 1.15 | 0.70, 1.88 | 1.27 | 0.66, 2.47 | 1.54 | | 0.94, 2.53 |
| *P-trend* | 0.30 |  | 0.11 | |  | | 0.02 |  |  | 0.67 |  | 0.58 |  | 0.05 | |  |
| **Model 2^b^** |  |  |  | |  | |  |  |  |  |  |  |  |  | |  |
| Q2 | 1.07 | 0.62, 1.82 | 1.38 | | 0.60, 3.16 | | 1.10 | 0.60, 2.01 |  | 1.07 | 0.64, 1.78 | 0.91 | 0.44, 1.87 | 0.87 | | 0.49, 1.52 |
| Q3 | 1.24 | 0.74, 2.06 | 1.11 | | 0.49, 2.52 | | 1.10 | 0.62, 1.96 |  | 0.95 | 0.57, 1.60 | 0.53 | 0.24, 1.17 | 0.72 | | 0.41, 1.27 |
| Q4 | 1.13 | 0.67, 1.90 | 1.29 | | 0.58, 2.87 | | 1.36 | 0.78, 2.37 |  | 1.17 | 0.71, 1.93 | 1.20 | 0.61, 2.36 | 1.54 | | 0.94, 2.54 |
| *P-trend* | 0.58 |  | 0.70 | |  | | 0.26 |  |  | 0.63 |  | 0.70 |  | 0.06 | |  |
| **Model 3^c^** |  |  |  | |  | |  |  |  |  |  |  |  |  | |  |
| Q2 | 1.10 | 0.64, 1.89 | 1.45 | | 0.63, 3.34 | | 1.20 | 0.65, 2.21 |  | 1.03 | 0.62, 1.71 | 0.92 | 0.44, 1.90 | 0.89 | | 0.51, 1.57 |
| Q3 | 1.35 | 0.81, 2.27 | 1.28 | | 0.56, 2.90 | | 1.26 | 0.71, 2.25 |  | 0.98 | 0.58, 1.65 | 0.56 | 0.25, 1.25 | 0.79 | | 0.44, 1.40 |
| Q4 | 1.11 | 0.66, 1.88 | 1.37 | | 0.61, 3.07 | | 1.50 | 0.85, 2.63 |  | 1.07 | 0.64, 1.77 | 1.09 | 0.54, 2.17 | 1.51 | | 0.91, 2.51 |
| *P-trend* | 0.59 |  | 0.61 | |  | | 0.15 |  |  | 0.84 |  | 0.94 |  | 0.08 | |  |
| Abbreviations: AMI, acute myocardial infarction; CI, confidence interval; CV, cardiovascular; HR, hazard ratio; Q, quartile; wt%, percentage by weight | | | | | | | | | | | | | | |  |  |
| a Adjusted for ADMA (µmol/L) | | | |  |  | |  |  |  |  |  |  |  |  | |  |
| b Adjusted for age, sex and ADMA (µmol/L) | | | | | |  |  |  |  |  |  |  |  |  | |  |
| c Adjusted for age (years), sex, ADMA (µmol/L), current smoking (yes/no), diabetes mellitus (yes/no), effective statin dose at discharge (0-6), extent of significant coronary artery stenosis (0-3) and eGFR (mL/min) | | | | | | | | | | | | | | | | |
